# Different Outcomes According to Needling Point Location Used in Sham Acupuncture for Cancer-Related Pain: A Systematic Review and Network Meta-Analysis

**DOI:** 10.3390/cancers15245875

**Published:** 2023-12-17

**Authors:** Boram Lee, Chan-Young Kwon, Hye Won Lee, Arya Nielsen, L. Susan Wieland, Tae-Hun Kim, Stephen Birch, Terje Alraek, Myeong Soo Lee

**Affiliations:** 1 KM Science Research Division, Korea Institute of Oriental Medicine, Daejeon 34054, Republic of Korea; qhfka9357@kiom.re.kr; 2 Department of Oriental Neuropsychiatry, Dong-Eui University College of Korean Medicine, Busan 47227, Republic of Korea; beanalogue@naver.com; 3 KM Convergence Research Division, Korea Institute of Oriental Medicine, Daejeon 34054, Republic of Korea; hwlee@kiom.re.kr; 4 Department of Family Medicine & Community Health, Icahn School of Medicine at Mount Sinai, New York, NY 10029, USA; arya@guasha.com; 5 Center for Integrative Medicine, University of Maryland School of Medicine, Baltimore, MD 21201, USA; lswieland@gmail.com; 6 Korean Medicine Clinical Trial Center, Korean Medicine Hospital, Kyung Hee University, Seoul 02447, Republic of Korea; rockandmineral@gmail.com; 7 Kristiania University College, School of Health Sciences, 0317 Oslo, Norway; sjbirch@gmail.com (S.B.); terje.alrak@uit.no (T.A.); 8 Department of Community Medicine, Faculty of Health Sciences, National Research Center in Complementary and Alternative Medicine (NAFKAM), Institute of Health Sciences, 9037 Tromsø, Norway

**Keywords:** acupuncture therapy, cancer pain, cancer-related pain, placebo, sham acupuncture

## Abstract

**Simple Summary:**

When sham acupuncture was set as a control in previous acupuncture clinical trials, the sham procedure was conducted either at the same points that were used for the verum acupuncture group or at nonindicated points. The network meta-analysis according to needling point location used in sham acupuncture for cancer-related pain revealed no significant difference in pain severity between verum acupuncture and sham acupuncture using the same points as verum acupuncture. However, verum acupuncture significantly improved the pain severity compared to sham acupuncture at sham points. Sham acupuncture needling at the same points as verum acupuncture is not a true placebo due to the lack of consideration for acupuncture point specificity.

**Abstract:**

Numerous acupuncture studies have been conducted on cancer-related pain; however, its efficacy compared to sham acupuncture remains controversial. We confirmed whether the outcome of acupuncture differs according to the needling points of sham acupuncture for cancer-related pain. We searched 10 databases on 23 May 2023 to screen acupuncture trials using sham acupuncture or waiting list as controls for cancer-related pain. Sham acupuncture was classified into two types, depending on whether the needling was applied at the same locations as verum acupuncture (SATV) or not (SATS). A network meta-analysis (NMA) was performed on the basis of a frequentist approach to assess pain severity. Eight studies (*n* = 574 participants) were included in the review, seven of which (*n* = 527 participants) were included in the NMA. The pain severity was not significantly different between SATV and verum acupuncture, but verum acupuncture significantly improved pain severity compared to SATS. The risk of bias affecting the comparisons between the verum and sham acupuncture was generally low. Previous acupuncture trials for cancer-related pain showed differing outcomes of sham and verum acupuncture, depending on the needling points of sham acupuncture. The application of SATV cannot be considered a true placebo, which leads to an underestimation of the efficacy of verum acupuncture.

## 1. Introduction

Sham acupuncture has been used as a control intervention in randomized controlled trials (RCTs) to test the efficacy of acupuncture therapy. The sham acupuncture used in previous trials was different from verum acupuncture in terms of needling techniques, such as the use of shallow needling or nonpenetrating sham acupuncture devices (e.g., Park sham needle) [1,2,3]. However, to the best of our knowledge, no previous acupuncture trials using sham acupuncture as a control have tested the physiological inertness of sham acupuncture in advance [1]. Additionally, although acupuncture point specificity has been reported in several studies [4,5], in some acupuncture clinical trials, the same points were used in the sham and verum acupuncture groups [1,6]. Among the many factors responsible for the effects of acupuncture, in cases where the acupuncture points are uncontrolled, they cannot be regarded as an inert placebo control [7,8,9]. Therefore, evidence for the efficacy of acupuncture derived from such clinical trials may underestimate the effect of real-world acupuncture. In our previous network meta-analyses (NMAs), we found that the comparative effect of verum acupuncture differs, depending on the needling point of sham acupuncture for chronic nonspecific low back pain (CLBP) and knee osteoarthritis, which are representative pain conditions in which acupuncture has been frequently used in clinical settings and for which many acupuncture studies have been performed [10,11]. These results raised the question of whether a similar pattern would be observed in other conditions/diseases.

Pain is the most common cancer symptom and is experienced by 44.5% of patients with cancer [12]. International guidelines recommend nonpharmacologic interventions, such as acupuncture therapy, for cancer pain management due to their efficacy and safety [13], and partly due to the opioid crisis [14,15,16,17,18,19]. Indeed, many patients with cancer use effective nonpharmacologic therapies, such as acupuncture, for pain control [20,21,22]. Despite extensive acupuncture studies on cancer pain, the evidence is inconsistent, and definitive conclusions cannot be reached [23,24,25,26]. The main reason for this is the lack of high-quality research and the heterogeneity of effect sizes compared to the sham acupuncture control [27]. Therefore, similar to our previous studies on CLBP and knee osteoarthritis, in this NMA, we attempted to determine whether the outcomes of verum acupuncture compared to sham acupuncture on cancer-related pain varied according to the needling points of sham acupuncture.

## 2. Methods

The protocol of this study was registered in PROSPERO (CRD42023444281), and the study is reported according to the Preferred Reporting Items for Systematic Reviews and Meta-analyses (PRISMA) extension statement incorporating NMA [28].

### 2.1. Eligibility Criteria

(1) Population: Studies involving adult patients with cancer-related pain without limitations on age, sex, race, or nationality were included. The cancer type and stage were not restricted. Cancer-related pain is a general term that refers to a variety of pain conditions, characterized by different etiologies, characteristics, and pathological mechanisms experienced by patients with cancer, for which there is still no standardized classification system [29]. Therefore, we included studies involving patients with not only pain directly accompanying the development of cancer (malignancy), but also pain associated with cancer treatment, including chemotherapy, hormone therapy, radiation therapy, and surgery, referring to previous studies [23,30];

(2) Intervention and control group: We included verum acupuncture as the type of intervention, and sham acupuncture and waiting list groups as the types of control groups. As for the type of verum acupuncture, only penetrating manual acupuncture was included, while acupuncture accompanied by other stimuli, such as electrical stimulation and laser, was excluded. Sham acupuncture commonly uses techniques and devices with shallow or no insertion [1,6,31]. In our analysis, sham acupuncture was classified according to the needling points as follows: (1) SATV, defined as sham acupuncture therapy at the same acupuncture points as the verum acupuncture group; and (2) SATS, defined as sham acupuncture therapy at different (sham) points compared to the verum acupuncture group. The waiting list group was included to create a connected loop for the NMA as a reference control group;

(3) Outcome measure: The outcome of interest was pain severity, as measured by the Brief Pain Inventory (BPI) pain severity subscale, Visual Analog Scale (VAS), Numerical Rating Scale (NRS), or other validated outcome measures. If multiple pain scales were used in the included studies, the highest priority was given to BPI pain severity subscale. We used the earliest results after the completion of all treatment sessions as the time point for the unit of analysis;

(4) Study design: Only parallel-group RCTs published in peer-reviewed journals were included.

### 2.2. Information Sources and Search Strategy

The following English, Chinese, and Korean databases were searched on 23 May 2023, without limitation on publication language: MEDLINE, Embase, Cochrane Central Register of Controlled Trials (CENTRAL), Allied and Complementary Medicine Database (AMED), China National Knowledge Infrastructure (CNKI), Wanfang data, Chongqing VIP, Oriental Medicine Advanced Searching Integrated System (OASIS), Koreanstudies Information Service System (KISS), and Korean Medical Database (KMbase). The reference lists of eligible studies and related review articles, as well as clinical trial registries, were manually searched to identify any missing studies. Search strategies were established through consultation with acupuncture and systematic review experts by reviewing several previous studies. The full search strategies implemented in all databases and the search results are described in Supplementary File S1. 

### 2.3. Study Selection and Data Collection 

The bibliographic information of studies identified through the database search and other sources was imported into EndNote 20 (Clarivate Analytics, Philadelphia, PA, USA), and the titles and abstracts of the studies were reviewed using the automatic duplicate removal function. Subsequently, the full texts of the eligible studies were retrieved, and the final selection of the included studies was determined following a full text review. 

The following data were extracted from the included studies in pilot-tested Excel form: basic characteristics (first author, country, sample size, study setting, and funding source), population (mean age, sex, and cancer type), interventions (details of verum and sham acupuncture), outcome measures, and results. Study selection and data extraction were conducted independently by two researchers (BL and CYK), and any disagreement was resolved by discussion between them.

### 2.4. Risk of Bias Assessment

The risk of bias in the included studies was assessed using the Cochrane risk of bias tool [32]. The following domains were evaluated as “low”, “unclear”, or “high risk of bias” in individual studies: random sequence generation, allocation concealment, blinding of participants, personnel (acupuncture therapist), outcome assessor, incomplete outcome data, selective reporting, and other sources of bias. Other biases were evaluated by examining potential factors that could affect the study results, including differences in baseline characteristics between the two groups. One researcher (BL) evaluated the risk of bias of the included studies, another researcher (CYK) independently reviewed the results, and, in case of disagreement, consensus was reached through discussion.

### 2.5. Data Analysis and Synthesis

The main characteristics of the included studies were qualitatively summarized. In addition to conducting a pairwise meta-analysis on direct evidence using Review Manger 5.4 (Cochrane, London, UK), an NMA based on the frequentist approach and consistency model was conducted on mixed evidence using network packages in Stata/MP 16 (StataCorp LLC, College Station, TX, USA). In both the pairwise meta-analysis and the NMA, a random-effects model was selected, considering inevitable clinical heterogeneity in acupuncture treatment methods between studies. Before performing the NMA, transitivity was checked, and statistical inconsistency was tested using the node-splitting method (local approach) and the design-by-treatment interaction model (global approach). The number of related direct RCTs and participants of each intervention included in the NMA was expressed as the line thickness and node size of the network map. Quantitative synthesis estimates are presented as an interval plot and league table. The statistical significance and directionality of the pairwise meta-analysis and NMA results were compared through the league table. Because pain severity is a continuous variable and was assessed using various questionnaires in the included studies, effect estimates were pooled using standardized mean differences (SMDs) with 95% confidence intervals (CIs). To rank interventions for cancer-related pain, the surface under the cumulative ranking curve (SUCRA) was calculated and expressed as 0–100%, where the higher the number, the better the pain improvement. We planned to assess the potential publication bias using a funnel plot and Egger’s test for asymmetry if 10 or more studies were included in the analysis. 

### 2.6. Certainty of Evidence Assessment

The certainty of the direct, indirect, and network evidence for findings was assessed by the Grading of Recommendations Assessment, Development, and Evaluation (GRADE) guidance as very low, low, moderate, and high, considering the risk of bias, indirectness, inconsistency, imprecision, and publication bias [33]. The imprecision domain was only assessed in the certainty of network estimates [33].

## 3. Results

### 3.1. Study Selection and Characteristics

**Figure 1:**
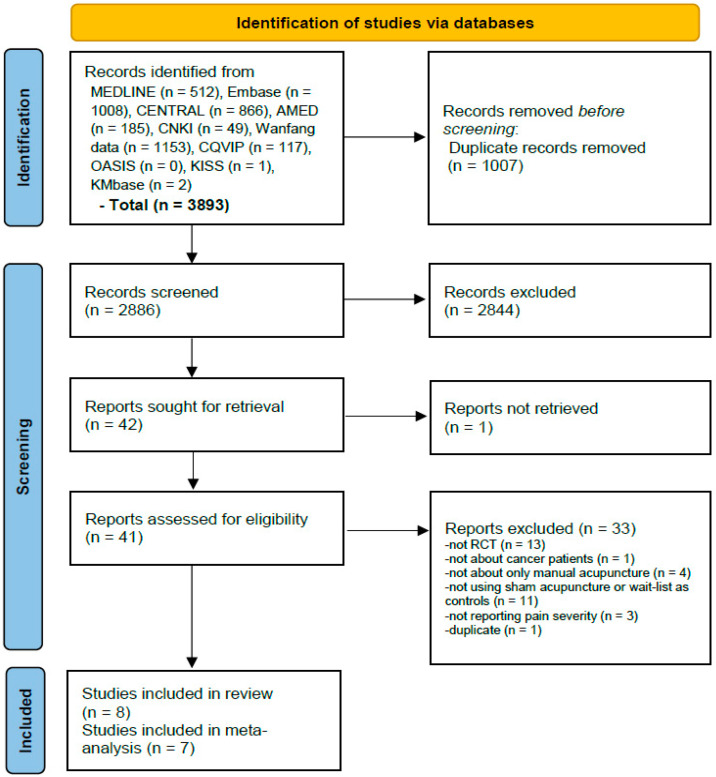
Flow diagram of the literature screening and selection processes. RCT, randomized controlled trial.

A total of 3893 studies were retrieved from the database searches. After deduplication, title and abstract review, and full text retrieval, the full texts of 41 studies were reviewed. Finally, 8 studies comprising 574 participants [34,35,36,37,38,39,40,41] were included in the systematic review after excluding 33 studies for the following reasons: not RCT (13 studies), not about patients with cancer (1 study), not about only manual acupuncture (4 studies), not using sham acupuncture or waiting list as controls (11 studies), not reporting pain severity (3 studies), and duplicate (1 study) (Supplementary File S2). In two of these studies [35,39], the pre- and post-treatment results were presented only as plots, with no presentation of estimates. An estimate for one study [39] was received through contact with the corresponding author, while another study, for which there was no response, was excluded from the meta-analysis [35]. In one study [34], only the mean and range values of the VAS on pain severity were reported, and the range was converted to standard deviation using the (maximum − minimum)/4 formula [42]. Therefore, 7 studies comprising 527 participants [34,36,37,38,39,40,41] were included in the pairwise meta-analysis and NMA (Figure 1).

**Figure 2:**
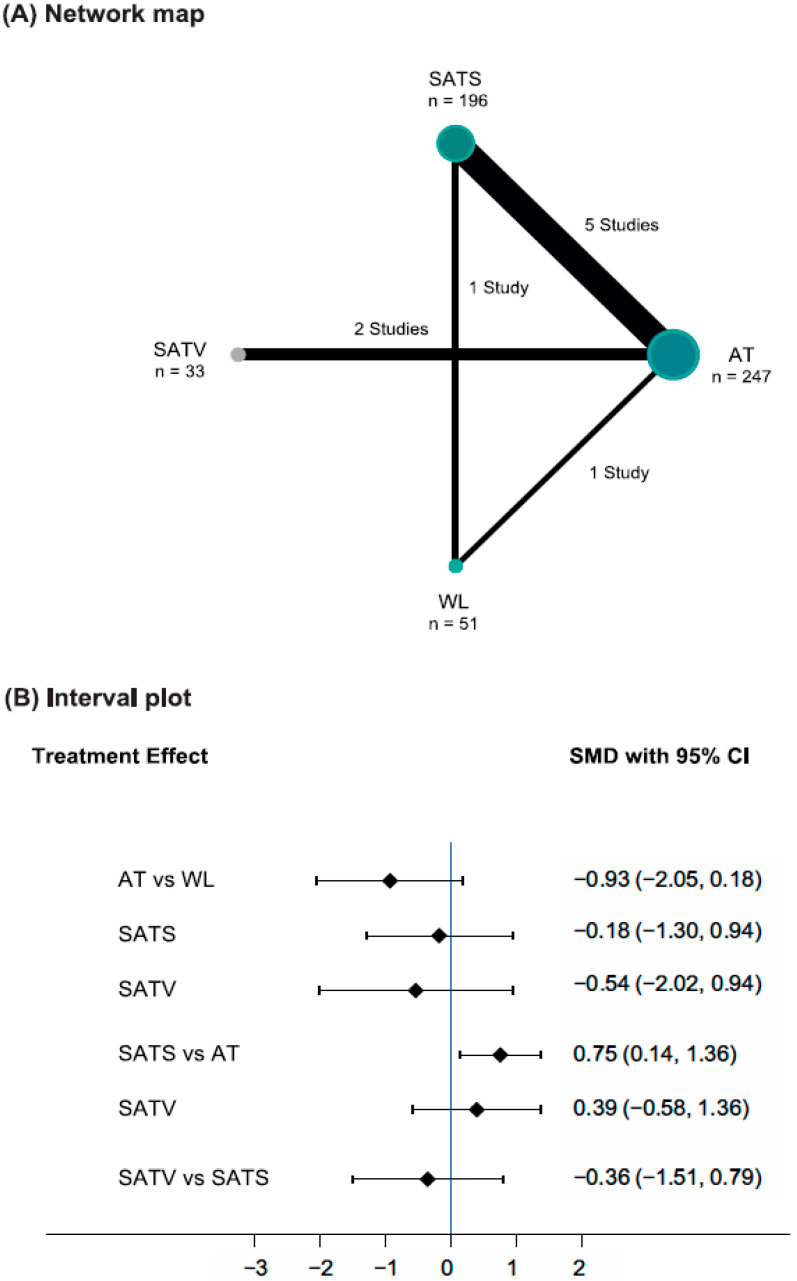
Network map and interval plot. The number of direct clinical trials and participants of each intervention included in the network meta-analysis were expressed as the line thickness and node size of the network map. In the interval plot, when the confidence interval does not pass 0 and is biased toward the left (right), the left (right) group is more effective than the other group in terms of improving pain severity. Abbreviations: AT, Acupuncture therapy; CI, Confidence interval; SATS, Sham acupuncture therapy at different points compared to the acupuncture group; SATV, Sham acupuncture therapy at the same acupuncture points as the acupuncture group; SMD, Standardized mean difference; WL, Waiting list.

Seven studies [34,35,36,37,38,40,41] were conducted on patients with pain induced by cancer treatment, such as surgery, hormone, or chemotherapy, and one study [39] was conducted on patients with malignancy-related pain. One study [38] compared verum acupuncture, sham acupuncture, and waiting list, and the remaining studies compared verum and sham acupuncture. As for the needling techniques of sham acupuncture, one study [35] used a nonpenetrating Park sham needle [3], and one study [36] performed superficial needling. Three studies [37,39,41] used nonpenetrating dummy studs or adhesive tape. One study [38] used shallow needling for the body and nonpenetrating adhesive tape without pellets for the ear. In one study [40], a minimum of five auricular needles were inserted in the ears in the verum acupuncture group at relevant points, whereas two auricular needles were placed in the ears at irrelevant points in the sham acupuncture group. In one study [34], sham acupuncture was divided into two groups, one group in which sterile steel implants were embedded at non-acupuncture points and another in which auricular seeds were affixed with tape at non-acupuncture points. Therefore, the effect sizes of the two sham acupuncture groups were combined and used for analysis. When classified according to the needling points of the sham acupuncture group, six studies [34,35,36,37,38,40] performed SATS and two studies [39,41] performed SATV. As for the pain severity outcome measure, a 0 to 100 VAS was used in two studies [34,35], the BPI–short form (BPI–SF) was used in two studies [36,38], and a 0 to 10 NRS was used in three studies [39,40,41]. In one study [41], pain at rest and movement-evoked pain severity were evaluated on a 0 to 10 NRS scale. Although assessing pain intensity during activity is also important, given that it is common to assess pain intensity during inactivity or after activity [43], for consistency with other studies, we included pain severity at rest in the analysis, as this was judged to fit the research question after discussion among researchers. One study [37] used both the BPI and 0 to 10 NRS, and the BPI was included in the analysis in accordance with our prioritization of outcomes (Table 1 and Supplementary File S3). A four-node network map was constructed (Figure 2A), and the contribution matrix for direct comparison with the NMA estimates is shown in Supplementary File S4. The *p*-value was 0.3975 in the global approach for inconsistency, and the *p*-value was >0.05 in the local approach, satisfying the statistical consistency assumption (Supplementary File S5).

### 3.2. Risk of Bias Assessment

An appropriate random sequence generation method was used in all studies; however, two studies [35,40] were evaluated as having an unclear risk of bias because they did not report information on allocation concealment. For three studies [34,38,41], blinding of participants was not possible, and, because the results were evaluated through patient self-reported questionnaires, blinding of outcome assessment was also evaluated to have a high risk of bias. In all studies, it was impossible to blind the acupuncturist. However, because pain intensity was measured by patient self-assessment, it was judged that it was unlikely to have affected the study results. One study [40] was evaluated as having a high risk of attrition bias because 8 out of 31 enrolled participants dropped out due to deaths or withdrawals, and no intent-to-treat analysis was performed. Two studies [35,36] showed differences in baseline characteristics between the verum and sham acupuncture groups; therefore, both were evaluated as having a high risk of other bias (Supplementary File S6).

### 3.3. Data Analysis: Pain Severity

The NMA revealed no significant difference in pain severity between the SATV and verum acupuncture arms (SMD: 0.39, 95% CI: −0.58 to 1.36). However, verum acupuncture significantly improved pain severity after treatment compared to SATS (SMD: −0.75, 95% CI: −1.36 to −0.14). The difference between SATV and SATS was not statistically significant, although the effect estimate favored SATV (SMD: −0.36, 95% CI: −1.51 to 0.79). Despite a significant pain improvement in the verum acupuncture and SATS groups compared to the waiting list in the pairwise meta-analysis, there was no significant difference in the NMA (Table 2 and Figure 2B). For pain improvement, the ranking based on SUCRA was the highest at 90.7% for verum acupuncture, followed by SATV (57.1%), SATS (29.9%), and waiting list (22.3%) (Supplementary File S7). It was not possible to evaluate publication bias using the funnel plot and Egger’s test because only seven studies were included in the NMA. 

### 3.4. Certainty of the Evidence

The certainty of direct and indirect evidence of individual comparisons for pain improvement was moderate to downgrading due to the risk of bias. The certainty of network evidence was moderate to low, further downgraded because of imprecision due to wide confidence intervals in some comparisons (Supplementary File S8).

## 4. Discussion

Previous acupuncture trials for cancer-related pain have shown inconsistent results compared to sham acupuncture [23,24,25,26]; therefore, we examined whether the needling points of sham acupuncture were related to the outcome of verum and sham acupuncture, as in our previous analyses of CLBP and knee osteoarthritis [10,11]. Through a comprehensive database search, 8 RCTs involving 574 participants were included in the systematic review, and 7 RCTs comprising 527 participants were included in the pairwise meta-analysis and NMA. In some acupuncture clinical trials, the acupuncture points used for the verum acupuncture group were used as the needling points for the sham acupuncture group; this cannot be used as a placebo control, given the lack of consideration for acupuncture point specificity [1,4,5,6]. Instead, the design is a comparison of the effectiveness of acupuncture techniques, except for the selection of acupuncture points, rather than a clinical trial testing acupuncture efficacy. The sham treatment in this design is a modified form of acupuncture that can be clinically effective in its own right, rather than a true sham/placebo [44,45]. Therefore, if these clinical trials are inadvertently included in a systematic review and meta-analysis summarizing the efficacy of acupuncture, this can lead to an underestimation of treatment effects. 

According to the NMA for pain severity, there was no significant difference between the verum acupuncture and SATV arms; however, verum acupuncture significantly improved pain severity compared to SATS. The statistical significance and direction of the effect size between the verum and sham acupunctures were consistent in pairwise and NMA. Interestingly, this result is consistent with our previous NMAs of CLBP and knee osteoarthritis, which were conducted with the same hypothesis [10,11]. Meanwhile, in the previous study on CLBP [10], SATV showed a significant improvement in pain and back-specific function compared to SATS. However, there was no significant difference in cancer-related pain between the two groups, although SMD favored SATV. This may have been due to the number of studies and participants included in each study; for the NMA on CLBP, 10 RCTs involving 4379 participants were included [10], while only 7 RCTs involving 499 participants were included for cancer-related pain. The small number of participants included in the analysis may have affected the precision of the study, resulting in a wide confidence interval. Considering that the direction of SMD was toward SATV in this study, the two groups may have shown significant differences if additional related large-scale trials were to be conducted. Additionally, the type of population in each study may have had an impact. In particular, cancer-related pain appears in various types and stages of cancer, and pain is also induced by malignancy or cancer treatment, each of which is more or less difficult to treat [46]. Although there were no problems in the statistical consistency test for performing the NMA, clinical differences in these diseases and conditions may have affected the study results.

The risk of bias affecting the comparisons between the verum and sham acupuncture groups was generally low, with the exception that participant blinding was not possible in some studies, and outcome assessor blinding was not possible because of the use of patient self-reported assessment tools. 

This study has several limitations that warrant discussion. First, the relatively small number of studies and participants included in the analysis lowers the precision of the results and affects the certainty of the evidence. In addition, as the cancer type, cause of pain, accompanying treatment, and details of the acupuncture treatment method of the included studies were clinically heterogeneous, their influence on the study results cannot be excluded. We intended to perform further analyses on these factors, but subgroup analysis was not possible due to the small number of included studies.

Nevertheless, the results of the current study are consistent with those of previous CLBP and knee osteoarthritis studies [10,11], suggesting that the outcome of sham acupuncture may differ depending on the points needled or stimulated, which may have led to an underestimation of the efficacy of acupuncture in previous trials. In the future, it will be necessary to confirm whether this hypothesis is consistent not only in other pain conditions, but also in non-pain conditions. In addition, based on our series of studies, the use of SATV in acupuncture efficacy trials should be discontinued. Furthermore, the physiological inertness of sham acupuncture has not been clearly established, regardless of whether it is conducted at verum or non-indicated points. As the factors responsible for inducing the effects of acupuncture are complex [7], and considering that sham acupuncture has specific effects, leading to an underestimation of the efficacy of verum acupuncture [47,48,49,50], it is becoming increasingly important to determine whether a true placebo control group is possible and whether sham acupuncture is viable as a control method [1,6,26,51]. Indeed, for the development of clinical and insurance guidelines specifically for acupuncture, it has been suggested that the effectiveness evidence of pragmatic trials versus usual care is more important than that of sham acupuncture control [6,52]; this is a trend that is happening across healthcare systems [53,54]. Under these controversial circumstances, continuing sham acupuncture controlled clinical trials will not only fail to accurately evaluate the efficacy of acupuncture, but will also add to the confusion in the research community, ultimately affecting clinicians, patients, and policy makers through the eventual implementation of inaccurate clinical practice guidelines. 

## 5. Conclusions

Previous acupuncture trials for cancer-related pain have reported differing outcomes of sham and verum acupuncture, depending on the needling points of the sham acupuncture arms. Sham acupuncture needling at the same points as verum acupuncture cannot be considered a true placebo because there is no consideration for acupuncture point specificity, which leads to an underestimation of the efficacy of verum acupuncture. This type of trial is not a test of acupuncture efficacy, but rather a comparison of acupuncture techniques relative to the intensity of stimulation at the indicated acupuncture points.

## Figures and Tables

**Table 1:** Characteristics of the included RCTs.

Study ID (Country)	Study Design	Population	Total Sample Size (AT/SAT/WL)	Mean Age (y)	Sex (Male/Female)	Details of SAT	Outcome (Pain Severity)	Treatment Duration	Timepoint for Analysis
Alimi 2003 (France) [34]	a parallel-group RCT (AT vs. SAT)	- Chronic peripheral or central neuropathic pain arising after cancer treatment, prolonged for at least 1 month - 0–100 pain VAS ≥ 30 mm	87 (29/58/–)	57 (range: 37–84)	17/70	SATS, Group 1 (28 participants): steel implants at non-acupuncture points; Group 2 (30 participants): auricular seeds fixed at non-acupuncture points	0–100 VAS	2 months	2 months
Bao 2013 (United States) [35]	a parallel-group RCT (AT vs. SAT)	- Postmenopausal women with early stage breast cancer, experiencing aromatase inhibitor-associated musculoskeletal symptoms - 0–100 pain VAS ≥ 20 mm	47 (23/24/–)	AT: median 61 (range: 45–85), SAT: median 61 (range: 44–82)	0/47	SATS, nonpenetrating Park sham needle at non-acupuncture points	0–100 VAS	8 weeks	8 weeks (not analyzed in meta-analysis)
Crew 2010 (United States) [36]	a parallel-group RCT (AT vs. SAT)	- Postmenopausal women with breast cancer, experiencing aromatase inhibitor-associated musculoskeletal pain - BPI–SF worst pain ≥ 3 points	38 (20/18/–)	Median 58 (range: 37–77)	0/38	SATS, superficial needling at non-acupuncture points	BPI–SF pain severity	6 weeks	6 weeks
Deng 2008 (United States) [37]	a parallel-group RCT (AT vs. SAT)	- Patients with cancer scheduled for unilateral thoracotomy	106 (52/54/–)	AT: median 65 (IQR: 58–72), SAT: median 63 (IQR: 57–70)	52/54	SATS, dummy studs without needle at non-acupuncture points	BPI pain severity, 0–10 NRS	ST36, shenmen: 1 week, others: 4 weeks	30 days
Hershman 2018 (United States) [38]	a parallel-group RCT (AT vs. SAT vs. WL)	- Postmenopausal or premenopausal women with early-stage breast cancer who were taking an aromatase inhibitor - BPI–SF worst pain ≥ 3 points	206 (101/54/51)	60.7 ± SD 8.6	0/206	SATS, shallow needling (body), or ear pellet with pellets removed (ear) at non-acupuncture points	BPI–SF pain severity	12 weeks	12 weeks
Kim 2018 (Republic of Korea) [39]	a parallel-group RCT (AT vs. SAT)	- Patients with advanced cancer who were being administered analgesics for cancer pain	27 (14/13/–)	56 (range: 42–73)	11/16	SATV, nonpenetrating bent needle at acupuncture points	0–10 NRS	3 weeks	3 weeks
Ruela 2018 (Brazil) [40]	a parallel-group RCT (AT vs. SAT)	- Patients with cancer receiving chemotherapy - 0–10 pain NRS ≥ 4 points	23 (11/12/–)	AT: 58.27 ± SD 10.09, SAT: 52.08 ± SD 7.99	5/18	SATS, penetrating auricular needle at irrelevant acupuncture points	0–10 NRS	8 weeks	9 weeks
Wang 2022 (China) [41]	a parallel-group RCT (AT vs. SAT)	- Diagnosed with gastric cancer by pathology and underwent open radical gastrectomy (operation time ≤ 3 h)	40 (20/20/–)	AT: 63.10 ± 8.30, SAT: 65.60 ± 6.10	21/19	SATV, nonpenetrating adhesive tape without needle at acupuncture points	0–10 NRS	6 days (embedded into the skin 24 h before the surgery, and was replaced once every 3 days)	5 days post-operation

AT, Acupuncture therapy; BPI, Brief pain inventory; BPI–SF, Brief pain inventory–short form; IQR, Interquartile range; NRS, Numeric rating scale; RCT, randomized controlled trial; SD, Standard deviation; SAT, Sham acupuncture therapy; SATS, Sham acupuncture therapy at different points compared to the acupuncture group; SATV, Sham acupuncture therapy at the same acupuncture points as the acupuncture group; VAS, Visual analog scale; WL, Waiting list.

**Table 2:** League table for pairwise meta-analysis (right upper part) and network meta-analysis (left lower part) effect estimates.

**WL**	**−0.71 (−1.05, −0.36)**	**−0.42 (−0.80, −0.03)**	**-**
−0.93 (−2.05, 0.18)	**AT**	**0.73 (0.19, 1.28)**	0.42 (−0.07, 0.92)
−0.18 (−1.30, 0.94)	**0.75 (0.14, 1.36)**	**SATS**	-
−0.54 (−2.02, 0.94)	0.39 (−0.58, 1.36)	−0.36 (−1.51, 0.79)	**SATV**

Results are presented as the standard mean difference (95% confidence interval). Comparison must be read from left to right. A standardized mean difference greater than zero indicates that the treatment on the left is favored in both pairwise and network meta-analyses. The values in bold text indicate statistical significance. AT, Acupuncture therapy; SATS, Sham acupuncture therapy at different points compared to the acupuncture group; SATV, Sham acupuncture therapy at the same acupuncture points as the acupuncture group; WL, Waiting list.

## Data Availability

The authors confirm that the data supporting the findings of this study are available within the article and its supplementary materials.

## Supplementary Materials

The following supporting information can be downloaded at: https://www.mdpi.com/article/10.3390/cancers15245875/s1, File S1: The search strategy used in each database; File S2: Excluded studies after full text review; File S3: Details of acupuncture treatment method and funding sources of the included studies; File S4: Contribution matrix; File S5: Results of testing inconsistency at the local level through the node splitting method; File S6: Risk of bias summary for all included studies; File S7: SUCRA plot; File S8: The certainty of the evidence.
