# Supplementary material for: Different Outcomes According to Needling Point Location Used in Sham Acupuncture for Cancer-Related Pain: A Systematic Review and Network Meta-Analysis
Source: Cancers (Basel). 2023 Dec 17;15(24):5875. doi: 10.3390/cancers15245875 (PMC10741764; doi:10.3390/cancers15245875)
Supplement: Supplementary file 1 [file cancers-15-05875-s001.zip › cancers-2683589-SI.pdf]

Supplementary Online Contents

Supplement 1. The search strategy used in each database

Supplement 2. Excluded studies after full-text review

Supplement 3. Details of acupuncture treatment method and funding sources of the included studies

Supplement 4. Contribution matrix

Supplement 5. Results of testing inconsistency at the local level through the node splitting method

Supplement 6. Risk of bias summary for all included studies

Supplement 7. SUCRA plot

Supplement 8. The certainty of the evidence

Supplement 1. The search strategy used in each database

MEDLINE via PubMed

	Searches	Results
#1	Neoplasms[MH] OR Carcinoma[MH] OR cancer*[TIAB] OR neoplasm*[TIAB] OR carcino*[TIAB] OR tumor*[TIAB] OR tumour*[TIAB] OR malignan*[TIAB]	5,059,815
#2	Pain[MH] OR Analgesia[MH] OR pain*[TIAB] OR analges*[TIAB] OR nocicept*[TIAB] OR neuropath*[TIAB]	1,204,295
#3	Acupuncture[MH] OR “Acupuncture Therapy”[MH] OR “Acupuncture Points”[MH] OR acupunct*[TIAB] OR acupoint*[TIAB] OR “Dry Needling”[MH] OR “dry needling”[TIAB] OR "filiform needle"[TIAB]	38,277
#4	“Randomized Controlled Trial”[PT] OR “Controlled Clinical Trial”[PT] OR randomized[TIAB] OR placebo[TIAB] OR “Clinical Trials as Topic”[Mesh:noexp] OR randomly[TIAB] OR trial[TI]	1,537,513
#5	animals[MH] NOT humans[MH]	5,121,735
#6	(#1 AND #2 AND #3 AND #4) NOT #5	**512**

Embase via Elsevier.com

	Searches	Results
#1	Neoplasm/exp OR Carcinoma/exp OR ‘malignant neoplasm’/exp OR cancer*:ab,ti OR neoplasm*:ab,ti OR carcino*:ab,ti OR tumor*:ab,ti OR tumour*:ab,ti OR malignan*:ab,ti	7,054,289
#2	Pain/exp OR Analgesia/exp OR pain*:ab,ti OR analges*:ab,ti OR nocicept*:ab,ti OR neuropath*:ab,ti	2,332,120
#3	acupuncture/exp OR acupuncture*:ab,ti OR ‘acupuncture point’/exp OR ‘body meridian’/exp OR ‘body meridian’:ab,ti OR acupoint*:ab,ti OR 'dry needling'/exp OR 'dry needling':ab,ti OR ‘filiform needle’:ab,ti	62,335
#4	'crossover procedure':de OR 'double-blind procedure':de OR 'randomized controlled	3,135,287

	trial':de OR 'single-blind procedure':de OR (random* OR factorial* OR crossover* OR cross NEXT/1 over* OR placebo* OR doubl* NEAR/1 blind* OR singl* NEAR/1 blind* OR assign* OR allocat* OR volunteer*):de,ab,ti	
#5	#1 AND #2 AND #3 AND #4	**1,008**

CENTRAL

	Searches	Results
#1	MeSH descriptor: [Neoplasms] explode all trees	110,900
#2	MeSH descriptor: [Carcinoma] explode all trees	17,997
#3	(cancer* OR neoplasm* OR carcino* OR tumor* OR tumour* OR malignan*):ti,ab,kw	259,820
#4	#1 OR #2 OR #3	271,680
#5	MeSH descriptor: [Pain] explode all trees	73,178
#6	MeSH descriptor: [Analgesia] explode all trees	11,724
#7	(pain* OR analges* OR nocicept* OR neuropath*):ti,ab,kw	258,565
#8	#5 OR #6 OR #7	269,263
#9	MeSH descriptor: [Acupuncture] explode all trees	713
#10	MeSH descriptor: [Acupuncture Therapy] explode all trees	6,380
#11	MeSH descriptor: [Acupuncture Points] explode all trees	2,494
#12	MeSH descriptor: [Dry Needling] explode all trees	158
#13	(acupunct* OR acupoint* OR “dry needling” OR "filiform needle"):ti,ab,kw	21,189
#14	#9 OR #10 OR #11 OR #12 OR #13	21,517
#15	(#4 AND #8 AND #14) in Trials	**866**

AMED via EBSCO

	Searches	Results
#1	SU Neoplasms OR TX cancer* OR TX neoplasm* OR TX carcino* OR TX tumor* OR	24,052

	TX tumour* OR TX malignant*	
#2	SU Pain OR SU Analgesia OR TX pain* OR TX analges* OR TX nocicept* OR TX neuropath*	41,878
#3	SU Acupuncture OR SU "Acupuncture Therapy" OR SU "Acupuncture Analgesia" OR SU Acupoints OR SU Needles OR SU Needling OR SU "Dry needling" OR TX acupuncture* OR TX acupoint* OR TX needl*	12,757
#4	#1 AND #2 AND #3	**185**

CNKI

	Searches	Results
#1	(SU='癌'+'肿瘤'+'瘤') AND (SU='痛') AND (SU='针')	**49**

Wanfang data

	Searches	Results
#1	(题名或关键词:癌 OR 题名或关键词:肿瘤 OR 题名或关键词:瘤) AND (题名或关键词:痛) AND (题名或关键词:针)	**1153**

Chongqing VIP

	Searches	Results
#1	M=(癌 OR 肿瘤 OR 瘤) AND M=(痛) AND M=(针)	**117**

OASIS

	Searches	Results
#1	(암|종양|신생물) (통증) (침)	**0**

KISS

	Searches	Results
#1	제목=(암|종양|신생물) AND 제목=(통증) AND 제목=(침)	**1**

KMbase

	Searches	Results
#1	(([TITLE=암] OR [TITLE=종양]) OR [TITLE=신생물])	9,525
#2	[TITLE=통증]	3,106
#3	[TITLE=침]	4,613
#4	#1 AND #2 AND #3	**2**

Supplement 2. Excluded studies after full-text review

- Not randomized controlled trials: 13

1. Acupuncture can ease radiation therapy pain for cancer patients. Chinese Health Care. 2011(9):75.
2. Gao F. Clinical experience in the treatment of cancer pain with acupuncture. Journal of Emergency in Traditional Chinese Medicine. 2007;16(8):1004-5.
3. Yan J, Zhao S, Lu M. Needling method(cultivate the spirit and promoting blood circulation to arrest pain)for treatment of cancer pain Journal of Changchun University of Traditional Chinese Medicine. 2014(1).
4. Li L. Clinical Observation on Acupuncture in Treating Abdominal Pain of Gastric Cancer. Journal of Clinical Acupuncture and Moxibustion. 2000(12):9.
5. Li X, Jia C. Observation on the efficacy of Ziwu flow acupuncture in the treatment of cancer pain in 118 patients. Chinese Journal of Rural Medicine and Pharmacy. 1994(3):32-3.
6. Xu S, Li H, Meng X, Xu M, Yang L. Clinical observation on 42 cases of gastric cancer pain treated with acupuncture at Zusanli point. Acupuncture Research. 1994(Z1):131.
7. Song F. 30 cases of uterine fibroids treated with balance acupuncture. Huaxia Medicine. 2001;005(4):43-4.
8. Wang S, Jiang X, Wang J. Clinical observation on 106 cases of liver cancer pain treated with auricular acupuncture. Journal of the Practical Chinese with Modern Medinine. 2005;18(5):732-.
9. Chu S, Xu M. Clinical observation on the application of acupuncture at Zusanli point for analgesia due to cancerous abdominal pain. Practical Clinical Journal of Integrated Traditional Chinese and Western. 1994;007(1).
10. Shu Z. Acupuncture can ease radiation treatment pain for cancer patients. Middle-aged and Elderly Health. 2011(5):5.
11. Liu K, Dong G, Fei Y. Traditional acupuncture relieves cancer pain. Family Medicine. 2022(11):31.
12. Zhang W. 160 cases of cancer pain treated with acupuncture. Journal of Emergency in Traditional Chinese Medicine. 2008;17(4):543-4.
13. Alimi D. L'acupuncture auriculaire avec des aiguilles semi-permanentes soulage la douleur chez les patients cancereux. Acupunct Moxibustion. 2005;4(2):147-52.

- Not about cancer patients: 1

1. Han XR, Yue W, Chen HC, He W, Luo JH, Chen SX, et al. Treatment duration of wrist-ankle acupuncture for relieving post-thyroidectomy pain: A randomized controlled trial. J Integr Med. 2023;21(2):168-75.

- Not about only manual acupuncture: 4

1. Tu J. Effect of Acupuncture on Analgesic Effect and Immune Function of Patients with Breast Cancer

During Perioperative Period. Zhejiang Journal of Traditional Chinese Medicine. 2018;53(12):899.
2. Peng H, Peng HP, Xu L. Exploring the Feasibility of Applying Double Blind Design in Randomized Controlled Clinical Trial of Acupuncture Treating Carcinous Pain——A randomized controlled double blind trial in a small sample. Journal of Jiangxi University of Traditional Chinese Medicine. 2010;22(4):26-31.
3. Wu J, Wang Y, Zhang Y, Zhao H. Clinical observation of acupuncture combined with auricular therapy and three-step analgesic ladder in treating cancer pain. Shanghai Journal of Traditional Chinese Medicine. 2017;51(10):48-51.
4. Wu J, Zhang Y, Zhao H, Wang Z, Shen K, Deng H, et al. Clinical study on acupuncture combined with auricular point therapy in the treatment of cancer pain. Chinese Journal of Traditional Chinese Medicine. 2014;32(8):1904-6,后插8.

- Not using sham acupuncture or wait-list as controls: 11

1. Jiang B, Chen F, Deng S, Du H. 25 cases of cancer pain treated with acupuncture of Taichong and Hegu. Zhejiang Journal of Traditional Chinese Medicine. 2016;51(4):270.
2. Guo Z, Guo G, Yang J, Shao Y. Effect of acupuncture treatment on pain and quality of life in patients with advanced gastric cancer. International Journal of Traditional Chinese Medicine. 2015(4):371-3.
3. Dan Y, Liang Y, Tao Y. Clinical study on the analgesic effect of acupuncture on cancer pain. Chinese Acuponcture & Moxibustion. 1998(1):17-8.
4. Dang W, Yang J. Clinical study on acupuncture in treating gastric cancer pain. Chinese Acupuncture and Moxibustion. 1995(5):277-80,60.
5. Xiao J, Li L. Observation on 50 Cases of Acupuncture Relieving Cancer Pain. Acupuncture Research. 1994;19(3).
6. Li Y, Jiang S, Li F, Han C. Research on the analgesic effect of acupuncture on patients with advanced tumors. Academic Periodical of Changchun College of Traditional Chinese Medicine. 1998;14(3):29.
7. Luo Z, Yan R, Zhu S, Huang G. Analysis of the efficacy of acupuncture at the second metacarpal holographic point in the treatment of severe pain in advanced cancer. Guangdong Medical Journal. 1992(2):94-5.
8. Bian R. Observation on the analgesic effect of acupuncture on cancer pain. Journal of Practical Traditional Chinese Medicine. 1999(7):28.
9. Hu X, Ling CQ, Zhou QH. Clinical observation on wrist-ankle acupuncture for treatment of pain of middle-late liver cancer. Chinese acupuncture& moxibustion [zhong guo zhen jiu]. 2004;24(3):149-51.
10. Xiao JH, Li LJ. Observation of 50 cases of cancer pain relieved by acupuncture. Zhen Ci Yan Jiu [acupuncture research]. 1994;19(3):139-40.
11. Zeng K, Dong H, Chen H, Chen Z, Li B, Zhou Q. Wrist-Ankle Acupuncture for Pain After Transcatheter Arterial Chemoembolization in Patients with Liver Cancer: a Randomized Controlled Trial. American journal of Chinese medicine. 2014;42(2):289-302.

- Not reporting pain severity: 3

1. Wang T, Chen Y, Lou G. Impact of Intradermal Needle Therapy on Pain after Transcatheter Arterial Chemoembolization Operation of Hepatocellular Carcinoma Patients. Journal of Traditional Chinese Medicine. 2015;56(23):2033-5,48.
2. Deng G. Acupuncture As a Potential Opioid-Sparing Pain Management Intervention for Patients Undergoing Cancer Treatment. Med Acupunct. 2020;32(6):394-5.
3. Deng G, Giralt S, Chung DJ, Landau H, Siman J, Search B, et al. Acupuncture for reduction of symptom burden in multiple myeloma patients undergoing autologous hematopoietic stem cell transplantation: a randomized sham-controlled trial. Supportive care in cancer. 2017:1-9.

- Duplicate: 1

1. Hershman DL, Unger JM, Greenlee H, Capodice J, Lew DL, Darke A, et al. Comparison of Acupuncture vs Sham Acupuncture or Waiting List Control in the Treatment of Aromatase Inhibitor-Related Joint Pain: A Randomized Clinical Trial. JAMA Netw Open. 2022;5(11):e2241720.

Supplement 3. Details of acupuncture treatment method and funding sources of the included studies

Study ID	Acupuncture protocol	Treatment points	Depth of insertion	Needle stimulation	Needle retention time	Needle type	Number of treatment sessions	Frequency	Other interventions	Qualification or experience of acupuncturists	Study setting	Funding sources
Alimi 2003	Individualized	Individualized ear acupuncture points where an electrical response had been detected	maximum 0.7 mm	NR	Individualized (until the dates needles or seeds fell out/off)	sterile steel implants with 3.4 mm long, 1.2 mm diameter and height	2	once a month	Analgesics as needed	a medical doctor/associate professor teaching auricular acupuncture at Paris XIII medical school (Bobigny, France)	Pain Management Unit at the Institut Gustave Roussy	grants from Institut Gustave Roussy (contrat de recherche clinique n°98-83), Schwa Medico, and the Lion's Club
Bao 2013	Standardized	CV4, CV6, CV12, bilateral LI4, PC6, GB34, ST36, KI3, BL65	0.5 inch	NR	20 min	0.25 x 40 mm needle with Park sham needle	8	once a week	None	NR	Sidney Kimmel Comprehensive Cancer Center at Johns Hopkins and the University of Maryland Greenebaum Cancer Center	ASCO Foundation Young Investigator's Award, Susan Komen Postdoctoral Fellowship Award, Breast Cancer Research Foundation, Maryland Affiliate of Susan G. Komen for the Cure Craft grant

Crew 2010	Semi-standardized	TE5, GB41, GB34, LI4, ST41, KI3, (ear) shenmen, kidney, liver, upper lung, sympathetic *Joint-specific points -knee: SP9, SP10, ST34 -fingers: SI5, SI3, EX-UE9, LI3 -lumbar: GV3, GV8, BL23 -shoulder: LI15, TE14, SI10 -hip: GB30, GB39 -wrist: TE4, LI5	proper needling depth as determined by standard point locations	De qi, restimulated manually once during each session	30 min	body: 25 or 40 mm and 34-gauge, ear: 15 mm and 38-gauge	12	twice a week	Nonnarcotic and nonsteroidal pain medications as needed	one United States–trained and New York State-licensed acupuncturist	NR	a Lance Armstrong Young Investigator Award and an Advanced Clinical Research Award from the American Society of Clinical Oncology with funding from AVON Products Foundation and the Breast Cancer Research Foundation
Deng 2008	Standardized	bilateral BL12 to BL19, extra point (Wei Guan Xia Shu), ST36, (ear) shenmen	NR	De qi	ST36, shenmen: 1 week, others: 4 weeks	~1.5 mm long with a diameter of ~0.2 mm	1	once	None	NR	Memorial Sloan Kettering Cancer Center	National Cancer for Complementary and Alternative Medicine grant AT002989
Hershman 2018	Semi-standardized	TE5, LI4, GB41, GB34, ST41, KI3, (ear) unilateral shenmen, kidney, liver, upper lung, and sympathetic *joint specific points -shoulder: LI15, TE14,	inserted at traditional depths	De qi, restimulated manually once during each session	30-45 min	body: 1 inch, 1.5 inches and 34-gauge or 3 inches and 30-gauge, ear: 15 mm and 38-gauge	18	twice a week for 6 weeks, followed by once a week for 6 weeks	None	licensed acupuncturists (at least 3000 hours) and medical doctor acupuncturists (at least 100 hours)	11 academic centers and clinical sites	National Institutes of Health (NIH) National Center for Complementary and Integrative Health and the Office of Research on Women's

		SI10<br>-wrist: SI5, TE4, LI5<br>-fingers: SI3, EX-UE9, LI3<br>-lumbar: GV3, GV8, BL23<br>-hip: GB30, GB39<br>-knee: SP9, SP10, ST34										HealthR01AT006376; NIH/NCI/DCP (Division of Cancer Prevention) grant UG1CA189974 and legacy grant U10CA37429.
Kim 2018	Semi-standardized	CV12, bilateral ST25, LI4, LR3, PC6, and 0-3 ashi points	NR	All patients were instructed to press all the needle sites with their hands twice a day	3 weeks (change every 48-72 hours)	0.18 × 1.3 × 1.5 mm (intradermal acupuncture needle)	1	once	Analgesics as needed	doctors of Korean medicine, at least 3 years of clinical experience, and had received more than 6 years of college education in Korean medicine	Dankook NGC Korean medicine clinic	Bio-Synergy Research Project (NRF-2017M3A9C4065964) of the Ministry of Science, ICT and Future Planning through the National Research Foundation; and the Comprehensive and Integrative Medicine Institute (CIMI), Daegu, Republic of Korea (Grant # CIMI-15-01-07)
Ruela 2018	Semi-standardized	(ear) unilateral shenmen, kidney, sympathetic, muscle relaxation, and the energy balance points (defined through the Five Elements theory)	NR	The manual stimulation of the needles was not recommended.	the needles remained fixed on the points for up to seven days	0.20 x 1.5 mm	8	once a week	Analgesics as needed	a researcher, who has the necessary certification, to guarantee the homogeneity of the technique	UNACON of the House of Charity Nossa Senhora do Perpétuo	Coordenação de Aperfeiçoamento de Pessoal de Nível Superior (CAPES)

											Socorro – Santa Casa of Alfenas	
Wang 2022	Standardized	(ear) unilateral shenmen, sympathetic nerve, thalamus, point zero, and omega 2	NR	Patients were instructed by the acupuncturists to press the acupuncture points three times a day, and when the NRS≥4, each point was pressed for two minutes at a time	6 days	0.2 x 0.6 mm	1	once	Analgesics as needed	a registered acupuncturist, with a master's degree in acupuncture and more than three years of clinical experience in acupuncture	the Affiliated Hospital of Nanjing University of Chinese Medicine	a research grant from Affiliated Hospital of Nanjing University of Chinese Medicine (Y19074), the Priority Academic Program Development of Jiangsu Higher Education Institutions (2019YSHL089)

NR, not recorded; NRS, numeric rating scale.

Supplement 4. Contribution matrix

Network meta-analysis estimates	Direct comparisons in the network: AvsB	AvsC	AvsD	BvsD
**Mixed estimates**				
AvsB	31.5		34.3	34.3
AvsC		100.0		
AvsD	17.5		65.0	17.5
BvsD	21.0		21.0	57.9
**Indirect estimates**				
BvsC	19.0	39.7	20.7	20.7
CvsD	9.6	45.2	35.6	9.6
**Entire network**	16.9	30.8	29.6	22.6
**Included studies**	5	2	1	1

A, acupuncture therapy; B, sham acupuncture therapy at different points compared with the acupuncture group; C, sham acupuncture therapy at the same acupuncture points as the acupuncture group; D, waiting list.

Supplement 5. Results of testing inconsistency at the local level through the node splitting method.

Side	Direct comparison		Indirect comparison		Difference		p value
	Coefficient	SE	Coefficient	SE	Coefficient	SE	
AT WL	0.6947427	0.6853752	1.993709	1.413889	-1.298967	1.564879	0.406
AT SATV	0.3930153	0.4953613	1.869405	446.529	-1.47639	446.5292	0.997
AT SATS	0.7516725	0.3117922	-0.7220365	452.9581	1.473709	452.9582	0.997
SATS WL	0.4291064	0.691101	-0.8700041	1.405537	1.299111	1.564906	0.406

Note. All the evidence about these contrasts comes from the trials which directly compare them.

AT, acupuncture therapy; SATS, sham acupuncture therapy at different points compared with the acupuncture group; SATV, sham acupuncture therapy at the same acupuncture points as the acupuncture group; WL, waiting list.

Supplement 6. Risk of bias summary for all included studies

	Random sequence generation (selection bias)	Allocation concealment (selection bias)	Blinding of participants (performance bias)	Blinding of acupuncture therapist (performance bias)	Blinding of outcome assessment (detection bias)	Incomplete outcome data (attrition bias)	Selective reporting (reporting bias)	Other bias
Alimi 2003	+	+	−	+	−	+	+	+
Bao 2013	+	?	+	+	+	+	+	−
Crew 2010	+	+	+	+	+	+	+	−
Deng 2008	+	+	+	+	+	+	+	+
Hershman 2018	+	+	−	+	−	+	+	+
Kim 2018	+	+	+	+	+	+	+	+
Ruela 2018	+	?	+	+	+	−	+	+
Wang 2022	+	+	−	+	−	+	+	+

Low, unclear, and high risk, respectively, are represented with the following symbols: "+", "?", and "-".

Supplement 7. SUCRA plot

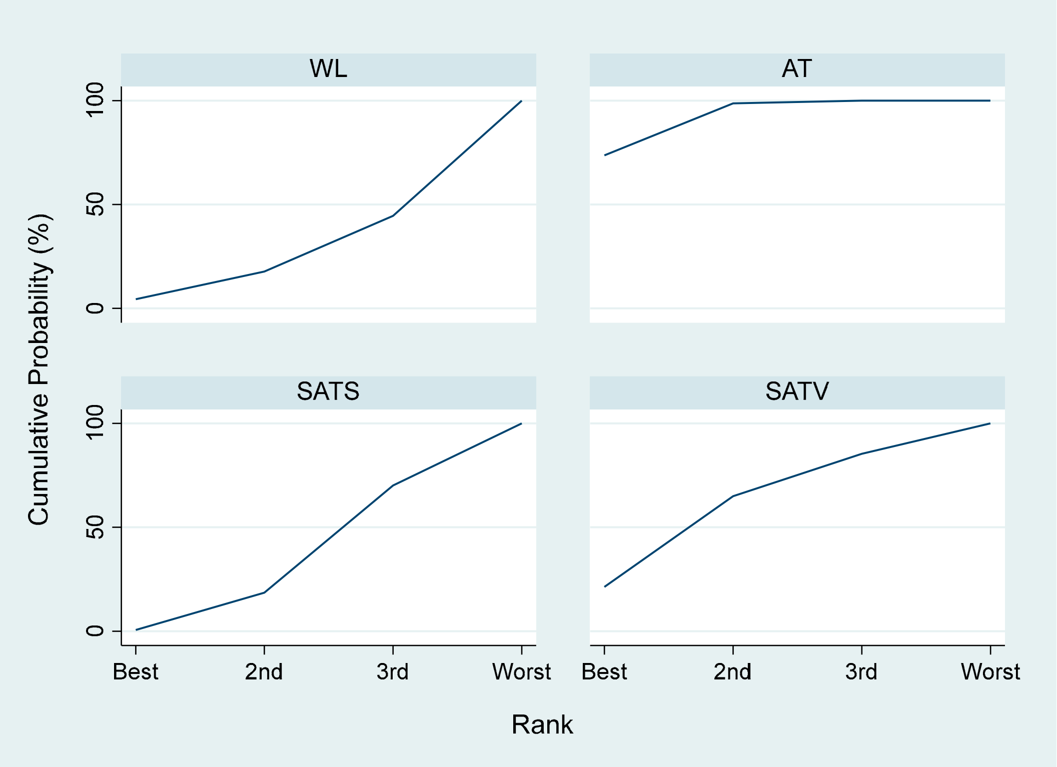

AT, acupuncture therapy; SATS, sham acupuncture therapy at different points compared with the acupuncture group; SATV, sham acupuncture therapy at the same acupuncture points as the acupuncture group; WL, waiting list.

*SUCRA is a numeric presentation of the overall ranking and presents a single number associated with each treatment. SUCRA values range from 0 to 100%. The higher the SUCRA value, and the closer to 100%, the higher the likelihood that a therapy is in the top rank or one of the top ranks; the closer to 0 the SUCRA value, the more likely that a therapy is in the bottom rank, or one of the bottom ranks.

Supplement 8. The certainty of the evidence

**Comparison**		**Direct evidence**	**Indirect evidence**	**Network meta-analysis**
AT	SATV	Moderate<br>Risk of bias (-1)	-	Low<br>Risk of bias (-1)<br>Imprecision (-1)
AT	SATS	Moderate<br>Risk of bias (-1)	Moderate<br>Risk of bias (-1)	Moderate<br>Risk of bias (-1)
AT	WL	Moderate<br>Risk of bias (-1)	Moderate<br>Risk of bias (-1)	Low<br>Risk of bias (-1)<br>Imprecision (-1)
SATV	SATS	-	Moderate<br>Risk of bias (-1)	Low<br>Risk of bias (-1)<br>Imprecision (-1)
SATV	WL	-	Moderate<br>Risk of bias (-1)	Low<br>Risk of bias (-1)<br>Imprecision (-1)
SATS	WL	Moderate<br>Risk of bias (-1)	Moderate<br>Risk of bias (-1)	Low<br>Risk of bias (-1)<br>Imprecision (-1)

AT, acupuncture therapy; SATS, sham acupuncture therapy at different points compared with the acupuncture group; SATV, sham acupuncture therapy at the same acupuncture points as the acupuncture group; WL, waiting list.
